# *In-vitro* red blood cell partitioning of doxycycline

**DOI:** 10.4103/0253-7613.56073

**Published:** 2009-08

**Authors:** P.V. Deshmukh, P.C. Badgujar, M.M. Gatne

**Affiliations:** Department of Pharmacology and Toxicology, Bombay Veterinary College, Mumbai - 400 012, India

**Keywords:** Doxycycline, partitioning, red blood cell

## Abstract

**Objective:**

*In-vitro* red blood cell (RBC) partitioning of doxycycline was studied to determine whether doxycycline penetrates RBC and its concentration was assayed keeping in view its high lipophilicity.

**Materials and Methods:**

Standardization of doxycycline was performed in whole blood and plasma of cattle by microbiological assay using *Bacillus subtillis* ATCC 6633 as indicator organizm. Actual concentration of the drug was obtained by comparing zone inhibition with standard graph and the extent of partitioning was mathematically calculated.

**Results:**

The R^2^ value of standard graph for doxycycline was 0.9934 and 0.9727 for plasma and whole blood, respectively. Overall, RBC partitioning of doxycycline was found to be 18.40 ± 1.70%.

**Conclusions:**

Overall RBC partitioning of doxycycline indicated low penetration into RBC. Plasma is the fluid suggested for pharmacokinetic evaluation of doxycycline.

## Introduction

High lipophilicity of certain drugs may increase the extent of RBC penetration and act as temporary storage of a drug and eventually affect the drug's *in-vivo* behavior. It cannot be-over-ruled that plasma drug concentration may get affected due to high penetration into RBCs. Therefore, it would be interesting to study the *in-plasma* profile of drugs such as doxycycline having high plasma protein binding as well as high lipophilicity. Such information will help choose the appropriate matrix among whole blood, plasma or serum for assaying pharmacokinetic behavior of the drug.[1] However, the significance of RBC partitioning is not really appreciated although it is stressed.

Tetracyclines supersede other antibiotics, spectrum wise, by virtue of action against haemoprotozoan infections like *Anaplasma, Theileria, Eherlichia* and *Malaria.* These protozoa, in due course of their life cycle, enter RBCs and such infected RBCs are the main source of infection to other animals via intermediate host.[2] In case of *Theileria*, micromerozoites enter RBC via ticks of *Rhipicephalus* and *Hyaloma* spp. In case of *Anaplasma,* blood sucking flies like *Tabanus* and S*tomoxys* and ticks act as intermediate host.[3] Even after clinical phase of disease some merozoites may remain in RBCs and disease may endure. The dormant stages of malarial parasite in RBCs may cause relapse of malaria. Little is known about drugs acting on erythrocytic stages of parasite.

Doxycycline is a member of the Tetracycline group derived semi synthetically. It is superior to its co members in having high lipophilicity, extensive protein binding,[4] improved antimicrobial spectrum and different pharmacokinetic profile. It has a five to 10 fold higher lipophilicity than oxytetracycline and chlortetracycline resulting in greater tissue penetration.[5]

*In-vitro* RBC partitioning of doxycycline was studied keeping in view its high lipophilicity as compared to oxytetracycline and tetracycline. This study is an effort to determine the extent of penetration of Doxycycline in RBCs.

## Materials and Methods

The standard drug Doxycycline Hyclate (British Pharmacopoeia, 2000) was procured from Wockhardt Pharmaceuticals, Mumbai. Whole blood from cattle was obtained from Deonar abattoir, Mumbai prior to slaughter through jugular vein with the help of sterile 18G needle using heparin as an anticoagulant. The concentration of the drug was estimated by microbiological assay[6] using *Bacillus subtillis* ATCC 6633, obtained from Food and Drugs Administration, Mumbai.

### Method of standardization

**Figure 1:**
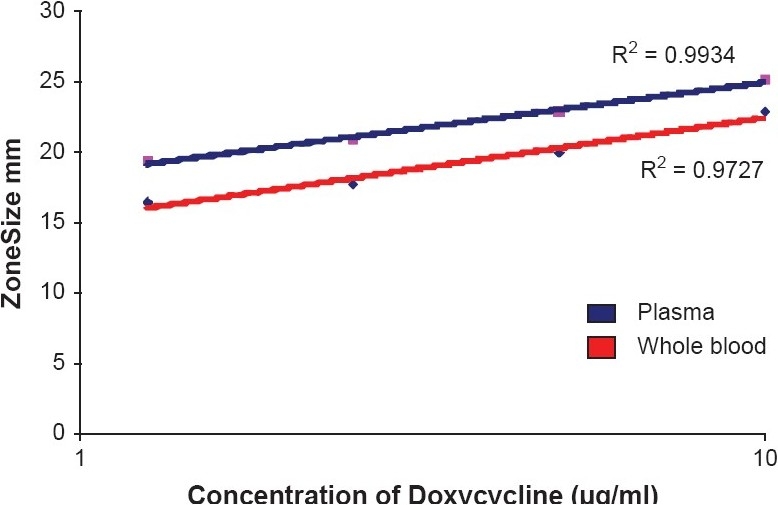
Standard graph of Doxycycline [Concentration verses zone size (diameter in mm)]

Standard concentrations of doxycycline were prepared in whole blood and plasma of cattle. *Bacillus Subtillis* culture prepared in normal saline; density adjusted to 25% transmittance at 580 nm[7] was used. About 0.1 ml of culture was added in 175 ml of Muller Hington Agar (Himedia lab Ltd.) and poured on leveled microbial assay plate. Equidistant wells were punched in agar after its solidification. Standard drug was serially diluted at concentration 10, 5, 2.5 and 1.25 μg/ ml and 100 μl of each and added to these wells in triplicates. After incubating the plate for 12 hours, zones of inhibition were measured using zone reader scale (Himedia Ltd.) and the mean zone size was recorded. Standard graph of concentration (X axis) versus mean zone size (diameter in mm - Y axis) was plotted for whole blood and plasma [Figure 1].

### Red blood cell partitioning

To study RBC partitioning, serial dilutions (10, 5, 2.5 μg/ml) of doxycycline were performed in whole blood (6 ml) of which PCV was measured after collection and incubated for 24 hours at 37°C allowing sufficient time for drug to penetrate RBCs. After incubation, plasma and RBCs were separated by centrifugation of four ml whole blood at 1200 rpm for 10 minutes. About 100 μl of remaining whole blood (after incubation), plasma, RBC pack and standard dilutions (to ensure performance of the set-up) each were added in punched wells in triplicates and a zone of inhibition was observed after 12 hours. The concentration of drug was estimated with the help of a standard graph and average from triplicate was drawn. The procedure was repeated three times to eliminate errors.

### Extent of partitioning

Concentration estimated with the help of standard graph was considered as observed and based on which concentration in plasma and RBC was calculated using its PCV. Percentage of RBC penetration was calculated based upon this value.

$\text{Calculated concentration in RBC}=\frac{\text{Observed concentration in RBC}\times \text{PCV\%}}{100}$

$\text{Calculated concentration in Plasma}=\frac{\text{Calculated concentration in Plasma}\times \text{Plasma \%}}{100}$

Further, extent of partitioning of doxycycline was calculated by using following formula:[8]

$\begin{array}{l} {\text{K}}_{\text{e/p}}=\frac{\text{Concentration of Doxycycline in RBC}}{\text{Concentration of Doxycycline in plasma}}\\ {\text{K}}_{\text{b/p}}\text{=}\frac{\text{Concentration of Doxycycline in Whole Blood}}{\text{Concentration of Doxycycline in plasma}} \end{array}$

Where, K_e/p_: Erythrocyte to plasma concentration ratio; K_b/p:_ Whole blood-to-plasma concentration ratio

## Results

**Figure 2:**
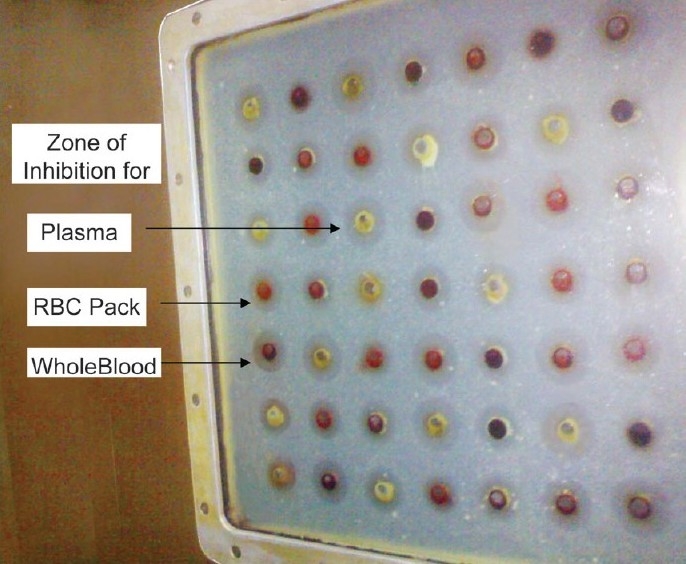
Microbiological assay plate showing zone of inhibition for whole blood, plasma and red blood cell

R^2^ value of standard graph for doxycycline was 0.9934 and 0.9727 for plasma and whole blood, respectively. PCV of whole blood was 43%. Microbiological assay plate showing zones of inhibition for whole blood, plasma and RBC pack is shown in [Figure 2]. Overall, RBC partitioning of doxycycline was found to be 18.40 ± 1.70% indicating moderate penetration into RBC. *In-vitro* concentrations of doxycycline (mg/ml) in whole blood, plasma and RBCs following addition of different known concentration are depicted in Table 1. K_e/p_, K_b/p_ values and RBC partitioning of doxycycline at different known concentrations is depicted in Table 2.

**Table 1:** *In-vitro* concentrations of doxycycline (μg/mls) in whole blood, plasma and red blood cell

*Concentration added (μg/ml)*	*Plasma Mean ± SE*	*Whole blood Mean ± SE*	*RBC Mean ± SE*
10	8.07 ± 0.88	7.19 ± 0.86	3.67 ± 0.67
5	3.70 ± 0.87	3.32 ± 0.32	1.42 ± 0.22
2.5	1.58 ± 0.22	1.27 ± 0.31	0.47 ± 0.17

**Table 2:** Red blood cell partitioning of doxycycline at different known concentrations

*Parameters*	*10 (μg/ml)*	*5 (μg/ml)*	*2.5 (μg/ml)*	*Overall mean*
K e/p	0.45	0.38	0.29	0.37
K b/p	0.89	0.90	0.80	0.86
Extent of penetration in red blood cell (%) (Mean ± SE)	21.62 ± 1.39	18.28 ± 1.54	15.51 ± 2.17	18.40 ± 1.70

## Discussion

Doxycycline is a drug having high lipophilicity and also high plasma protein binding. So in such a contrasting situation it would be interesting to study what course doxycycline will follow *in-vitro* so as to predict its behavior *in-vivo*.

This study was done with the hypothesis that doxycycline might be entering RBCs owing to its high lipophilicity and may help in arresting the development of intraerythrocytic stages of protozoan parasite, so as to eliminate infected stages of protozoa in RBCs responsible for the spread of disease to healthy population via different intermediate hosts.

Doxycycline is reported to have 92.3 ± 0.8% of protein binding.[9] Other tetracycline have no comparable plasma protein binding (Oxytetracycline - 18 to 22%, Chlortetracycline - 47 to 51% and Tetracycline - 31 to 41%)[10] and lipophilicity as doxycycline; therefore RBC partitioning, although low or moderate, cannot be compared with other co group members. However, most of the concentration remains in plasma, hence; the study suggests that plasma is the biological fluid to be collected for assay of the drug. However, in case of drugs with high plasma protein binding, consideration should be given to penetration into RBC as this may alter *in-vivo* behavior of drug. It was reported that for drugs with K_e/p_ or K_b/p_ larger than two in human subjects, measuring the concentration in whole blood or erythrocyte rather than plasma increases the sensitivity of an assay.[1] Considering this, 18.40 ± 1.70% partitioning obtained in the study is low with respect to its K_e/p_ and K_b/p_ values.

Further, RBC partitioning depends upon factors such as chemical nature of the drug, temperature, pH etc. RBCs may metabolize some of the drugs with the help of the enzymes present in it.[1] When considering assaying concentrations of drugs in whole blood, possible degradation by enzymes located in the RBCs must be excluded.[1] However, it was difficult to trace any reference whether doxycycline is metabolized by RBCs or not. The study revealed penetration into RBC but the method of uptake into RBC is yet to be explored. Further research may throw light on this aspect of the RBC. Hinderling reported that most of the drugs enter RBC by passive diffusion but lipophilicity was the single most important factor determining the extent of partitioning.

**Annexure:** 

*Pl no*	*Concentration*	*Plasma (μg/ml)*		*Red blood cell (μg/ml)*		*Whole blood (observed) (μg/ml)*	*Red blood cell %*
					
		Observed	Calculated	Observed	Calculated	Observed	
1	10	7.8	4.4	3.4	1.4	6.7	21.6
	5	3.7	2.1	1.2	0.6	3.4	15.2
	2.5	0.4	0.22	0.3	0.1	1.2	10.43
2	10	6.7	3.8	2.7	1.2	6.1	19.17
	5	2.2	1.3	1.2	0.5	2.8	18.54
	2.5	1.2	0.7	0.3	1.2	0.80	17.14
3	10	9.7	5.5	5.0	2.1	8.9	23.90
	5	5.2	3.0	1.9	0.79	3.85	20.51
	2.5	1.2	0.7	0.8	0.3	1.9	18.37
Total							18.31

$\begin{array}{l} \text{Calculated concentration in Plasma}=\frac{\text{Observed concentration in plasma}\times \text{Plasma \%}}{100}\\ \text{Calculated concentration in RBC}=\frac{\text{Observed concentration in plasma}\times \text{PCV \%}}{100} \end{array}$

In future, *in-vitro* erythrocytic protozoan culture can be prepared and the effect of treatment of various anti-haemoprotozoan drugs on erythrocytic stages could be revealed. At the same time various drugs having anti-haemoprotozoan activity can be assayed with such culture especially antimalarial drugs. Also, species variation in RBC partitioning of doxycycline can be assayed so as to use it in treatment against erythrocytic stages of haemoprotozoan infections. This will help consider RBC as a compartment in studying kinetic behavior of the drug.

This study confirms the use of plasma as a milieu for pharmacokinetic analysis of doxycycline and focuses on further need of research in RBC partitioning so as to reaffirm pharmacokinetic calculations.
